# Caffeinated beverage consumption among adolescents in Sagamu, Nigeria: implications for health promotion

**DOI:** 10.11604/pamj.2022.41.202.31696

**Published:** 2022-03-14

**Authors:** Oluwafolahan Sholeye, Olamide Akinyemi, Bankole Oyewole

**Affiliations:** 1 Department of Community Medicine and Primary Care, Olabisi Onabanjo University, Sagamu Campus, Ogun State, Nigeria,; 2 Department of Community Medicine and Primary Care, Olabisi Onabanjo University Teaching Hospital, Sagamu, Ogun State, Nigeria

**Keywords:** Caffeine, energy, drink, adolescents, school

## Abstract

**Introduction:**

the mental and physical stimulating effects of caffeine have led to an increase in consumption of caffeinated beverages. Adolescents are at an increased risk of excessive caffeine consumption and its associated adverse health consequences. This study therefore assessed the pattern of caffeinated drink consumption among in-school adolescents in Sagamu, Ogun State, Nigeria.

**Methods:**

a descriptive, cross-sectional study was carried out among 350 adolescents in Sagamu Township, selected via multistage sampling. Data were collected using a semi-structured self-administered questionnaire and analyzed with SPSS version 20.0. Relevant descriptive and inferential statistics were calculated with level of significance (p) set at <0.05.

**Results:**

respondents' mean age was 14.49 ± 1.37 years; 60.2% of respondents were male. Over 90% of respondents consumed caffeinated beverages; 19.2% consumed greater than 3 cans in a day; 67.8% always felt a strong urge to consume caffeinated drinks. Reasons for consumption include: to aid personal study (64.4%), thirst (47.1%), performance enhancement (34.1%), alertness (30.6%) and hunger (17.7%). Reported side effects include: nervousness (40.4%); mood swings (16.5%); palpitations (30.1%); insomnia (51.6%).

**Conclusion:**

consumption of caffeinated beverages was high among adolescents in Sagamu. Adequate caffeine control measures, with behavior change communication, will help to address this public health challenge among adolescents.

## Introduction

In recent times there has been a global drive for increased awareness of diet-related non-communicable diseases. This is partly because of the magnitude of the challenges associated with these chronic diseases and also due to the readily available and inexpensive preventive measures. Poor dietary habits, increased consumption of sugar-sweetened beverages and effect of excess caloric intake are documented among adolescents and young adults [1]. In addition to obesity, cardiovascular diseases, stroke and some cancers, caffeine intoxication is a main contributor to the morbidity and mortality associated with non-communicable and chronic diseases [2]. Excessive caffeine ingestion has become of great concern in several climes due to the forms and concentrations in which it occurs particularly as a major ingredient in a variety of drinks including sugar-sweetened beverages [3]. Caffeinated beverages vary in form, taste and volume in almost all regions of the world. They include sodas and carbonated drinks, energy drinks, coffee, caffeinated tea and some others mixed with alcohol. A common characteristic is that they contain a significant amount of caffeine [4]. Available evidence from literature show that consumption of these drinks occurs across age-groups and socio-economic status, but with preponderance among young person´s [5,6].

There has been a consistent increase in the consumption of caffeinated beverages among adolescents and young persons in many developed and developing countries. The amount consumed varies widely but a consistent finding is that many adolescents are consuming above the recommended (safe) level of caffeine [7,8]. Researchers in Ghana reported a high proportion of the population consuming caffeine in energy drinks, with as many as 70% of respondents consuming one to three cans per day [9]. In Canada and the United States, caffeinated beverage consumption has increased over time to a level of public health importance [10]. High levels of consumption of caffeinated drinks have been reported among adolescents and young persons in Slovakia [11], Poland [12], Saudi Arabia [13], Benin and Nigeria [14]. This has led to growing public health concerns about the increasing popularity of caffeinated beverages among adolescents and youth around the world along with the associated deleterious effects on human health especially in countries with lax regulations [7,15].

Consequences of high caffeine intake among adolescents go beyond acute phase physiological responses and may have long-term psychosocial and developmental implications [16]. Several researchers have reported an increased risk of substance use and other behavioral problems in addition to negative school outcomes among regular and heavy consumers of some caffeinated drinks. Excessive use of caffeine has been associated with anxiety, cardiac conduction anomaly, restlessness, insomnia and gut dysfunction [11,15,17]. Many adolescents are easily influenced by peers, media icons and adverts. Producers of many caffeinated drinks target adolescents and young persons via youth-focused advertisements and promotions, often portraying these drinks as healthy and energizing choices [6]. Energy drinks are many times aggressively advertised as supplying unusual strength, alertness, elation and sexual prowess [9,16].

Adolescence is generally considered a healthy phase of life for most people and a period of experimentation with various lifestyle choices. However, several unhealthy behaviors, including negative eating attitudes, imbibed during this period may last for a long time with negative health consequences [18,19]. The school environment sometimes inadvertently provides access to caffeinated drinks, when it should actually be a place for health education and promotion. A previous study among in-school adolescents in Southwestern Nigeria reported a high prevalence of consumption of sweetened beverages including sodas and energy drinks, some of which were purchased on the school premises [20]. Adolescents in Sagamu, like their counterparts in other parts of the world, are exposed to well designed and targeted advertisement without public enlightenment on the negative consequences of high levels of caffeinated beverage consumption. This study therefore determined the pattern of caffeinated beverage consumption among in-school adolescents in Sagamu, Southwestern Nigeria.

## Methods

The study was carried out in Sagamu Township, an urban area comprising of several settlements, educational facilities, including nursery and primary schools as well as the state-owned tertiary hospital with its associated college of health sciences. It is home to people of various ethnic groups and diverse occupations, majority being traders and civil servants. The descriptive cross-sectional study was carried out between October and December 2018 at selected public secondary schools in Sagamu. Adolescents attending public secondary schools, who were fully resident in Sagamu Township, with no history of a chronic medical condition, constituted the study population. The sample size was determined using the Leslie and Kish formula for descriptive studies [21], using a prevalence of 75% for caffeinated drink consumption from a previous study [22]. A sample size of 350 was calculated allowing for 20% non-response.

Multi-stage sampling was used for selection of study participants. Four out of six wards were selected from the township by simple random sampling. One public secondary school was selected from each pre-selected ward. Probability proportionate to size was used to determine the number of participants from each school. The students were stratified according to their levels of study following which proportionate allocation was carried out and systematic sampling was used for final selection of respondents. Data collection was done with the aid of a self-administered, semi-structured questionnaire. The questionnaire was designed specifically for the study following review of literature and the adaptation of previously validated questionnaires while taking the specific characteristics of the study population into account [6,12,23]. The instrument was then pretested at another town with similar characteristics as the study location. Relevant adjustments were made to the study instrument prior to data collection. Two trained research assistants were involved in data collection. Each questionnaire was assessed for completeness. Data analysis was done using statistical package for the Statistical Package for the Social Sciences IBM SPSS 21.0. Descriptive statistics were calculated and presented as frequencies, proportions, means and standard deviation. Chi square test was used to determine association between categorical variables, with the level of significance (p) set at <0.05.

Ethical clearance was obtained from Ogun State Ministry of Education, Science and Technology, Abeokuta as well as the health research ethics committee of the state Ministry of Health. The ethical approval reference number is OOUTH/HREC/432/2021. Official approval was granted by the Zonal Education Office, Sagamu and the respective school principals. Written informed consent and assent were obtained from study participants and their parents/guardians as necessary. Participation was fully voluntary and respondents were free to withdraw from the study at any given time. Strict confidentiality was ensured, while observing the principles of the Helsinki declaration.

## Results

The mean age of respondents was 14.4 ± 1.4 years; 60.2% were male; 51.8% were Muslims; 59.4% were of the Yoruba ethnic group and 54.6% had fathers with at least secondary level education. Majority (90.1%) of respondents were aware of caffeinated drinks. Forms of caffeinated drinks known to study participants were as follows: carbonated drinks (80.4%); alcoholic drinks (40.1%); coffee (70.1%); energy drinks (51.3%). Perceived side effects of caffeinated drinks were: anxiety (9%); insomnia (11.8%); addiction (25.7%); hallucination (22.8%); vomiting (26.6%); headache (48.5%); indigestion (27%); dehydration (45.5%); muscular pain (11.8%) and; palpitation (5.1%).

Almost all (92.0%) respondents consumed caffeinated drinks. Of these, 75.4% consumed coffee; 90.3% drank caffeinated tea; 95.4% drank soft drinks; 70.9% of respondents consumed energy drinks. Among those aged 13-15 years, 91.4% consumed caffeinated drinks; while 95.2% of respondents aged 16-18 years were consumers. There was no association (p=0.38) between age and consumption of caffeinated beverages. About a quarter (24%) consumed caffeinated drinks occasionally; 25.5% consumed them on a weekly basis; 29.4% consumed caffeinated beverages almost three or more times a week; while 21% consumed them daily. The consumption pattern had been on for less than a year among 10.3% of respondents; one year among 18.6% respondents; and more than a year in 72.1% of respondents. More male respondents (92.8%) consumed caffeinated drinks than females (91.2%), with no significant difference between them (p=0.84). All respondents who participated in sporting activities consumed caffeinated drinks compared with 78.6% of those who did not take part in any sporting event. There was a significant difference between both groups of students (p=0.004).

Majority of study participants (41%) consumed only one can (350 mls) of caffeinated drinks at once; 22% consumed two cans (700 mls) on each occasion; 19.2% drink more than three cans at any time they chose to consume caffeinated drinks. About a quarter (26%) preferred bottles to cans; 10% had no preference (Table 1). Reasons for consuming caffeinated drinks were: alertness (30.6%); aid personal study (64%); improvement of physical performance (34.1%); thirst (47.1%); hunger (17.7%); while at social gatherings (47.6%). Respondents´ choices of caffeinated beverages were influenced by: friends (27.4%); advertisement (25.3%); taste (24.4%); availability (4.1%); price (8.2%); stimulant effect (10.6%). Only 20.5% of respondents felt excited after every can of caffeinated beverages; 15% had increased the amount consumed to get the desired effect. A strong urge for caffeinated drinks was experienced by 67.8% of respondents; 44.4% mixed them with alcohol; 30.4% mixed their drinks with tramadol and other drugs; 49% skipped meals after consuming caffeinated drinks.

**Table 1:** pattern of respondents’ consumption of caffeinated beverages

Variable	Frequency	Proportion
**Consume caffeinated drinks**	**N=350**	
Yes	322	92.0
No	28	8.0
Type of drinks consumed*	**N=322**	
Coffee	264	75.4
Caffeinated tea	316	90.3
Sodas	307	95.4
Energy drinks	248	70.9
**Preferred package**	**N=322**	
Cans	205	63.7
Bottles	84	26.0
No preference	33	10.2
**Regularity**	**N=322**	
Daily	68	21.1
Thrice or more weekly	95	29.4
Weekly	82	25.5
Occasionally	77	24.0
**Mixed with alcohol**	**N=322**	
Yes	143	44.4
No	179	55.6
**Mix with drugs**	**N=322**	
Yes	98	30.4
No	223	69.6

* Multiple options were allowed

Reported unwanted effects of caffeinated drink consumption include: nervousness (40.4%); irritability (19.6%); dizziness (4.7%); vomiting (32.0%); insomnia (51.6%); hallucination (31.4%); aggression (42.9%); palpitations (30.1%). Withdrawal symptoms were reported by 20.6% of respondents (Table 2).

**Table 2:** reported adverse effects of caffeinated beverage consumption

Adverse effects	Response	
	Yes (%)	No (%)
Nervousness	130 (40.4)	192 (59.6)
Irritable behavior	63 (19.6)	259 (80.4)
Mood swings	53 (16.5)	269 (83.5)
Hallucination	101 (31.4)	221 (68.6)
Aggression	138 (42.9)	184 (57.1)
Palpitation	97 (30.1)	225 (69.9)
Insomnia	166 (51.6)	156 (48.4)
Dizziness	15 (4.7)	307 (95.3)
Vomiting	103 (32.0)	219 (68.0)
Craving for more	72 (22.4)	250 (77.6)
Meal skipping	157 (48.8)	165 (51.2)

## Discussion

The increased consumption of caffeinated drinks among adolescents and young adults remains a major concern to many health workers in different climes, particularly as a result of the short- and long-term consequences of such behavior [3,23]. The proportion of respondents in this study, who consumed caffeinated beverages is very high and confirms research reports from other countries in which the pervasive nature of caffeinated beverage consumption had become of public health importance [8,13,24]. It is very similar to findings among Omani students where a prevalence of 97% was reported for caffeinated drink consumption. The slightly higher prevalence could be because of the older age and greater freedom of the Omani University study participants [25]. It is however, much higher than the proportions reported by previous researchers [12,23,26,27] in more affluent countries. This may be due to the availability of several other alternatives in those places.

Similar to findings from previous studies, a few more males consumed caffeinated drinks than females, even though there was no significant difference between them [9,11,15,18] which may be due to an increased propensity for adventure among adolescent boys than girls. The higher prevalence of caffeinated drinks consumption among older adolescents in this study is in agreement with findings from Switzerland [15], Slovakia [11] and Saudi Arabia [13]. Older adolescents have been reported to be more likely to experiment with caffeine and other substances than their younger counterparts [18]. The type of caffeinated drinks consumed is similar to reports from other studies and is reflective of the tendency for adolescents to opt for sugar-sweetened beverages [1,20,24]. It is in agreement with findings among Omani students where coffee, soda and tea were the preferred beverages in a study of caffeinated drink consumption [25]. The preference for soft drinks and soda in this study corroborates findings from a previous study among in-school adolescents in Benin, Southern Nigeria [14].

With three-quarters of study participants consuming caffeinated energy drinks in this study, public health concerns about its growing popularity and misuse among adolescents and young adults may not be unfounded [5,10]. This is similar to findings from Saudi Arabia [13], South Africa [6], Oman [25] and Canada [8], where the consumption pattern of caffeinated drinks, particularly energy drinks are described as heavy and worrisome. It is slightly higher than the proportions reported among Polish adolescents [12], young persons in Bangladesh [27] and high school students in Canada [22].

Similar to findings among Omani students and young people in Ghana, taste and stimulant effect were influencers of the choice of drinks consumed [9,25]. In addition to those factors, price and availability were also important to respondents in agreement with findings among young people in Saudi [13]. Adolescents and young persons from higher income households were found to have a higher proportion of energy drink consumption compared to their colleagues in a study conducted in South Africa [6]. The contributions of peer pressure and advertisement were evident in this study and are similar to findings from a previous survey in which friends and advertisement had the most influence on first trial of a caffeinated beverage [23]. It further buttresses the arguments of many researchers that caffeinated drinks, particularly energy drinks with or without alcohol, are seriously advertised to the public with a special appeal to adolescents and young person´s [6,10,28].

Without doubt, a major concern with caffeinated drinks is the risk of caffeine toxicity with its attendant effects. However, a fairly recent trend is the increasing consumption of alcohol-mixed caffeinated beverages, particularly energy drinks [5]. Over a decade ago, a study among medical students in Messina reported as many as 48.4% of energy drink consumers mixing it with alcohol, with over a third of respondents having consumed alcohol more than three times in the preceding month [29]. Due to the slightly older age of university students, adolescents were thought to be safe, since many countries had laws restricting sale and use of alcohol and other substances to adults. Research has however, demonstrated a tendency for adolescents to mix caffeinated beverages with alcohol in addition to a preference for commercially available ones [5,23]. The proportion of respondents who mixed their drinks with alcohol in this study is quite high and above findings from Turkey [23], Canada [18], Lebanon [26] and Switzerland [15]. This brings to the fore a new public health challenge which is fast becoming global in reach, but also not seriously considered by many stakeholders in the recent past [5]. The finding is a cause for concern, considering the fact that alcohol should not be sold to adolescents younger than 18 years old in Nigeria. If unchecked, such may predispose apparently healthy adolescents and young persons to adverse effects of not only excessive caffeine consumption but also alcohol toxicity [3]. Age-restriction warning labels on highly caffeinated drinks have been demonstrated to be sub-optimal in the actual control of caffeine intake by adolescents and other minors, partly because parents and other responsible adults in the society do not often monitor compliance with guidelines [28].

The frequency of caffeinated beverage consumption found in this study is slightly higher than that reported from Saudi Arabia [13] probably because of the highly-regulated and restricted social environment in many Saudi towns. It is however, much higher than proportions from previous studies conducted in various countries [23,26,27]. In contrast to findings from this study, researchers in Benin, Southern Nigeria, reported as high as 87% of adolescents consuming soft drinks including caffeine -containing sodas on a daily basis [14]. This may be an indication of the easy access adolescents have to caffeinated beverages of various forms and an enabling environment for caffeine addiction.

The volume consumed by almost a quarter of respondents is very high with likelihood that the safety limit for caffeine and alcohol may be exceeded [9]. This is particularly important considering the fact that many high-volume consumers of caffeinated beverages often mix them with alcohol, which may mask the depressant effects and other symptoms of alcohol intoxication, thereby predisposing many adolescents to alcohol dependence [29]. Even though this study did not test for association between substance use and caffeinated beverage consumption, having almost a third of adolescents mixing their drinks with a mild opioid (Tramadol) lends credence to the previously documented association between caffeinated drinks and substance use [5,15]. Binge drinking, sensation seeking, higher intensity of alcohol ingestion and substance use were reported among more frequent consumers of caffeinated energy drinks in Canadian secondary schools [18,22].

The well-established association between participation in sporting activities and caffeinated drinks consumption was also evident in this study [12,26]. The quest for performance enhancement and energy boost have made energy drinks in particular a popular beverage among physically-active adolescents and young adults despite its many adverse effects [8,23,26]. All avenues to reach those participating in sports and other forms of physical activity are effectively utilized by energy drink manufacturers through mainstream media and social media channels [6,28]. The large proportion of respondents who consumed these beverages for alertness and improved personal study capacity was far higher than findings from Bangladesh [27]. It may be an indication for educational interventions that address adolescents´ study habits and learning capacity so that dependence on caffeine is significantly reduced.

In agreement with previous findings in medical literature, cardiovascular and neurological side effects were experienced by respondents in this study [3,5,13]. Insomnia, palpitations, and nervousness were commonly reported in agreement with previous studies [8,23,27]. Behavioral and mental health challenges were reported by regular consumers of caffeinated beverages in agreement with previous studies. The respondents who experienced irritability, restlessness, anxiety and aggressive behavior following caffeinated beverage consumption, buttress evidence in existing medical literature of the association between regular intake of those beverages and psychosocial problems [5,27]. With about two-thirds of respondents experiencing cravings for caffeinated drinks and over 10% having to take more drinks to experience the desired excitement, it is very likely that dependence has set in as reported by previous studies [27,29].

Arguably, adolescents simply exhibit the values of the society in which they reside. It therefore becomes imperative for regulatory authorities to ensure that age-restrictions on certain beverages like alcohol are adhered to. Adequate sensitization of parents and guardians to the ills of excessive consumption of caffeinated drinks will be a step in the right direction. This will encourage parental involvement in the monitoring of adolescents´ compliance with set standards for drinks and other nutritional products [6,27]. Behavior change communication on the ills of excessive caffeine consumption, with or without alcohol, specifically targeted at adolescents and young persons will go a long way in achieving the desired reduction in caffeine intoxication and its associated effects resulting from excessive intake of caffeinated drinks [4]. Adolescents need to be empowered to take charge of their own health and wellbeing.

Presently in Southwest Nigeria, caffeinated beverages are sold everywhere without any form of restriction or regulation. Many schools have shops or stalls where these drinks can be bought. The school environment is one that can be easily regulated. Access to those drinks need to be addressed by the local health authority and the zonal education office. It remains a viable and ideal setting for health education and promotion activities regarding prevention of diet-related non-communicable diseases as well as other lifestyle related health conditions. Public health officials may need to passionately communicate the adverse effects of excessive consumption of caffeinated drinks, including the sugar-sweetened sodas, to the general public (particularly adolescents and young adults) with more vigor than the advertising world [1]. Media engagement needs to be deliberate, focused and consistent, in a language that is understood by adolescents and a package that appeals to them like the product advertisements [6,28].

The cross-sectional nature of this study does not allow for establishment of causality; however, it is evident that excessive and unregulated intake of caffeinated beverages in adolescence, can have negative effects on various organs and systems of the human body. Also, estimation of caffeinated beverage consumption was based on respondents´ report and may not have been accurate. However, this study provides an insight into the consumption pattern of caffeinated drinks among adolescents in secondary schools in Sagamu Township and the need for a multi-disciplinary approach to address this emerging public health challenge.

## Conclusion

Consumption of caffeinated drinks was very high, with a slight male preponderance. The volume and frequency of consumption were very high, with associated alcohol and substance use. Neurologic and behavioral side effects were reported among consumers. Targeted behavior change communication will go a long way in addressing this public health concern.

### What is known about this topic

- There has been a consistent increase in the consumption of caffeinated beverages among adolescents and young persons in many developed and developing countries hence prompting regulation that largely been put in place in developed world;
- High caffeine intake among adolescents results in acute phase physiological responses and may have long-term psychosocial and developmental implications.

### What this study adds

- The prevalence of caffeine consumption among adolescents in an African population and highlights the motivations and side effects of caffeine consumption;
- Data and evidence that is necessary to advocate for behavior change communication and regulation.
